# Supplementary material for: Two mouse models carrying truncating mutations in Magel2 show distinct phenotypes
Source: PLoS One. 2020 Aug 17;15(8):e0237814. doi: 10.1371/journal.pone.0237814 (PMC7430741; doi:10.1371/journal.pone.0237814)
Supplement: S1 Raw images — (PDF) [file pone.0237814.s008.pdf]

Fig 3A raw image

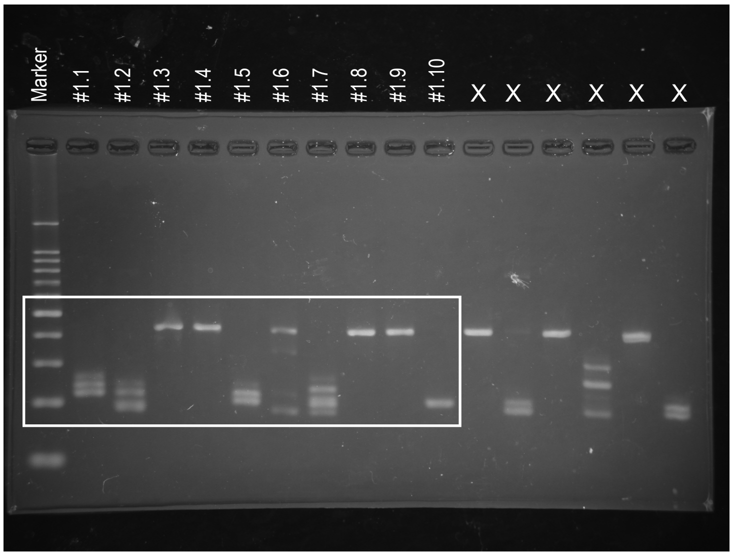

Fig 4A raw images

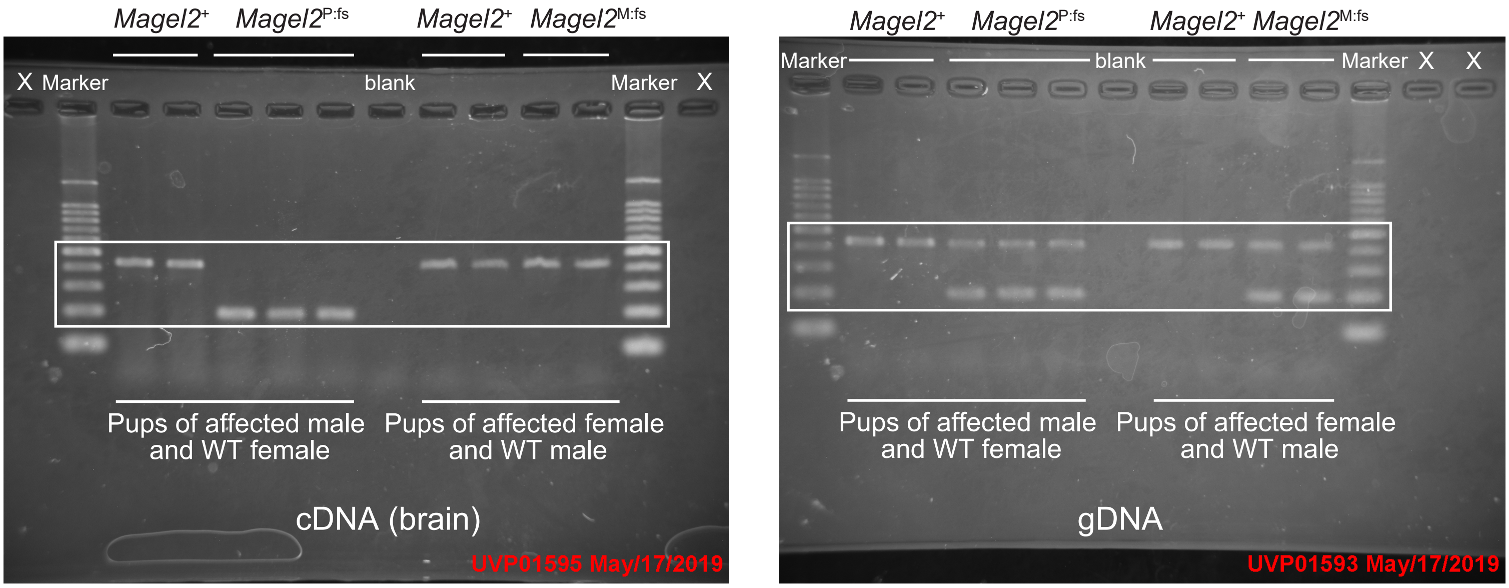

S6 fig raw image

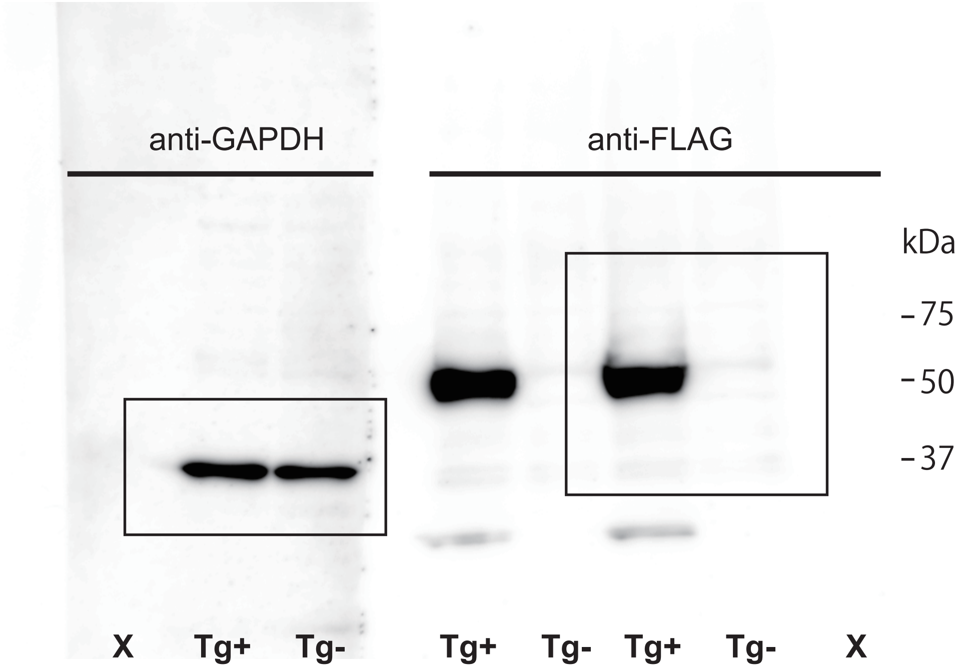

Tg+: HEK293 cell (transfected with pCAGGS1-Magel2-FLAG)
Tg- : HEK293 cell (untreated)
