# Supplementary material for: Two mouse models carrying truncating mutations in Magel2 show distinct phenotypes
Source: PLoS One. 2020 Aug 17;15(8):e0237814. doi: 10.1371/journal.pone.0237814 (PMC7430741; doi:10.1371/journal.pone.0237814)
Supplement: Attachment — Submitted filename: Response to Reviewers-1.docx [file pone.0237814.s009.docx]

Response to Editor

Thank you very much for giving me important comments. I agree with all seven comments and incorporated them to the manuscript (red color).

1. **Please ensure that your manuscript meets PLOS ONE's style requirements, including those for file naming.**

> We have incorporated this suggestion throughout our paper.

2. **Please provide additional information on the animal research and ensure you have included details on methods of sacrifice, methods of anesthesia and/or analgesia, and efforts to alleviate suffering."**
3. **Please include a comment about the state of the animals following this research. Were they euthanized or housed for use in further research? If any animals were sacrificed by the authors, please include the method of euthanasia and describe any efforts that were undertaken to reduce animal suffering.**

**>** We have provided additional information on Lines 185-186, 197-198, 204-205 and 215-216.

4. **PLOS ONE now requires that authors provide the original uncropped and unadjusted images underlying all blot or gel results reported in a submission’s figures or Supporting Information files.**

>We added Supporting Information file named ‘S1-raw-images’

5. **Please provide an amended statement that declares all the funding or sources of support (whether external or internal to your organization) received during this study.**

> There was no additional external funding received for this study. We added the description on Lines 448-449.

6. **I think that your study merits publication, I do not concur with your statement that “Our genome-edited mouse model could serve as a new model of truncated Magel2 to investigate the mechanism of SYS,” as there are no specific indications how the mechanisms in SYS should be addressed.**

> Thank you very much for your critical comment. We generally agree with you. We removed the above statement and added the new statement on Lines 60-62 and 442-444.

7. **Please amend your current ethics statement to confirm that your named ethics committee Institutional Care and Use Committee (IACUC) specifically approved this study.**

**>** We have provided additional information on Lines 189-190.

Responses to Reviewers

Red text indicates parts that were changed according to Editor. Blue indicates the parts that were changed according to Reviewer 1. Green indicates the parts that were changed according to Reviewer 2.

To Reviewer 1

Thank you very much for providing critical comments. We agree with all three comments and have incorporated the changes to the manuscript (blue text).

1. **The authors expressed the truncated version of Magel2 ubiquitously in all tissues, although Magel2 is normally expressed mainly in brain. Due to the small number of transgenic truncated specimens (n=3) the authors were unable to assess the patho-mechanism of the fetal or neonatal death, being due to either overexpression of the truncated protein or ubiquitous expression; i.e. this is a “no data experiment” since no conclusions can be drawn. The authors are strongly advised to closely investigate the patho-mechanism of fetal or neonatal death of the truncated version of Magel2, since this was the major objective in their study.**

> Thank you very much for your important comment. We completely agree with you. We tried to extract protein from the brain of dead mice in the overexpression model, and to detect truncated Magel2 protein with FLAG tag. However, we were not able to detect FLAG tag signal by western blot analysis because extracted proteins were denatured. Therefore, we were limited in our ability to investigate the patho-mechanism of fatal or neonatal death. We have added additional description in Lines 362-365, 429-431.

2. **The CRIPR-Cas generated frameshift Magel2 mice did not show obvious abnormalities in physical findings and failed to recapitulate the common phenotype of SYS. Their value in investigating pathophysiology in SYS is thus extremely limited. Other models for the effects of the more severe phenotype of Magel2 frameshift mutations should be considered and discussed.**

>Thank you very much for your critical comment. We generally agree with you. In humans, the severity of SYS depends on the specific location of truncating mutations; c.1996dupC and c.1996delC mutations show more severe phenotypes than other truncating mutations. We think our frameshift *Magel2* mice did not recapitulate common phenotype of SYS, but rather the mild phenotype of SYS. We have added additional description in Lines 413-419. We deleted the description ‘Therefore, our genome-edited mouse model could serve as a new model of truncated *Magel2* to investigate the mechanism of SYS’ on Lines 395-396 of the original manuscript. We also changed the description in Lines 442-444.

3. **The authors should cite relevant primary literature regarding PWS etiologies; it is common practice in recent years to cite reviews which again cite reviews and so on, but not mention the original publications.**

> We added description of the etiologies of PWS in Lines 75-77, and cited the original publications.

I believe that incorporating your advice has made the manuscript better. Thank you once again.

To Reviewer 2

Thank you very much for giving me good comments. I agree with all two comments and incorporated them to manuscript (green color).

1. **The decreased number of life-born pups with the frameshifting variant of Magel2 on the paternal allele may indicate prenatal lethality, as has been suggested for the Magel2-LacZ mouse. A power calculation should be done and subsequently that hypothesis should be tested.**

>Thank you very much for your important comment. We calculated the statistical power, and found it to be 0.186. Therefore, the power is low, and we can not reject the possibility that *Magel2*^P:fs^ mice show increased prenatal lethality. Nevertheless, the effect of the variant appears to be very low, and it would be necessary to increase the number of mice to more than 900 to obtain enough power, which is not feasible. We revised the sentence in Line 303, and added “power 0.186.” We also revised the sentence in Line 381, and added “although statistical power was not high.”

2. **Lines 87 and 390: MAGEL2 is misspelled.**

**>** I corrected the description.

I believe that incorporating your advices comments has made the manuscript better. Thank you once again.
